# Hyaluronic Acid Reduces Bacterial Fouling and Promotes Fibroblasts’ Adhesion onto Chitosan 2D-Wound Dressings

**DOI:** 10.3390/ijms21062070

**Published:** 2020-03-18

**Authors:** Ilaria Silvestro, Mariangela Lopreiato, Anna Scotto d’Abusco, Valerio Di Lisio, Andrea Martinelli, Antonella Piozzi, Iolanda Francolini

**Affiliations:** 1 Department of Chemistry, Sapienza University of Rome, Piazzale A. Moro, 5, 00185 Rome, Italy; ilaria.silvestro@uniroma1.it (I.S.); valerio.dilisio@uniroma1.it (V.D.L.); andrea.martinelli@uniroma1.it (A.M.); 2 Department of Biochemical Sciences, Sapienza University of Rome, Piazzale A. Moro, 5, 00185 Rome, Italy; mariangela.lopreiato@uniroma1.it (M.L.); anna.scottodabusco@uniroma1.it (A.S.d.)

**Keywords:** hyaluronic acid, chitosan, wound dressings, fibroblasts adhesion, *Staphylococcus epidermidis*

## Abstract

Wound healing is a dynamic process that can be seriously delayed by many factors including infectious complications. The development of dressings with intrinsic wound healing activity and/or releasing bioactive compounds may help with addressing such an issue. In this study, hyaluronic acid (HA) at different percentages (1–35%) was used to modify chitosan (CS) biological and physico-chemical properties in order to obtain 2D-matrices able to promote healing and protect from infection. HA incorporation in the CS matrix decreased film transparency and homogeneity, but improved film water uptake and surface wettability. The water vapor transmission rate (WVTR) increased up to a 5% HA content, where it reached the highest value (672 g/m^2^ day), and decreased for higher HA contents. At all of the tested HA concentrations, HA affected mechanical properties providing matrices more flexible than pure CS with benefit for wound care. Pure CS films permitted *S. epidermidis* adhesion and biofilm formation. That was not true for CS/HA matrices, where HA at concentrations equal to or greater than 5% was able to avoid *S. epidermidis* adhesion. Fibroblasts adhesion also took benefit from the HA presence in the film, especially at 5% content, where the best adhesion and proliferation was found.

## 1. Introduction

Wound dressings are widely used in clinical practice for standard wound management [1,2] and the performance of a dressing can regulate the success of the healing process. Although, a number of new material formulations have been proposed in the last decade [3,4], the research in this field is still very active since an ideal material able to meet the various and often specific dressings requirements in relation to the wound type has not yet been developed. In this regard, the main purposes of wound dressings are to provide a temporary physical protection from the external environment, to absorb wound exudates, as well as to confer gas permeability [5]. Indeed, in the absence of complications, wound healing is a natural process, which should bring to proper wound closure through different phases, namely hemostasis, inflammation, proliferation and remodeling [6]. However, different factors, such us microbial contamination of the wound bed and oxidative stress at the wound level as a consequence of diseases (diabetes and cardiovascular diseases), can slow down or hamper tissue regeneration processes (chronic wounds) [7]. There is, therefore, a clinical demand to develop novel antimicrobial materials [8,9] that, besides physically protecting the wound, could protect also from infection.

Polysaccharides, e.g., chitosan, alginate and hyaluronic acid, have been considered to be advantageous as wound dressing materials [10]. Indeed, they combine suitable hydrophilicity needed for wound exudates’ uptake, with transparency that is, instead, convenient for monitoring wound healing. Particularly, chitosan (CS), obtained by partial *N*-deacetylation of chitin, possesses important biological properties like biocompatibility, biodegradability and hemostatic activity [11,12]. In addition, being a cationic biopolymer, CS also shows intrinsic antibacterial capability versus a wide range of bacterial species especially gram positives [13,14]. Another advantage of chitosan is its easy processability that permits to obtain membranes [15,16], hydrogels [17], scaffolds [18], fibers [19] and nano- and microparticles [20]. This versatility promotes its application in a lot of biomedical applications including tissue engineering [21], drug delivery [22,23] and wound dressings [11,15]. Chitosan combination with other biopolymers or synthetic materials is widely used to modify CS mechanical and biological properties [24,25,26]. Specifically, chitosan combination with hyaluronic acid (HA), a linear polysaccharide composed by N-acetyl-glucosamine and d-glucuronic acid units, has lately emerged as a particularly advantageous strategy for wound dressings applications [27,28,29,30,31,32,33], since it can permit to combine the peculiar biological properties of these two biopolymers. Specifically, HA, being the main component of extracellular matrix (ECM), has been shown to influence several cellular events, such as attachment, migration and cellular proliferation, with benefits in terms of wound healing [34]. In addition, a potential protective role of HA versus bacterial adhesion has been recently reported [35], even if studies in this context are very few. Usually, relatively low amounts of HA (lower than 1%) have been used in the literature to develop chitosan/hyaluronic acid dressings and an antibacterial agent, often silver, has been also entrapped in these dressings in order to potentiate the dressing resistance to bacterial fouling [28,29,33]. 

In this framework, the aim of the present work was to prepare and characterize bi-dimensional chitosan/hyaluronic acid (CS/HA) matrices at high HA contents, ranging from 1% to 35%, in order to investigated if, in these conditions, HA could play the dual role of promoting wound healing and protecting wound from bacterial contamination, thus avoiding the use of antibacterial agents for preventive purposes. Particularly, the effects of HA on CS/HA matrix physical properties (morphology, wettability, water uptake, water vapor transmission rate and mechanical properties) as well as on matrix ability to stimulate *in vitro* human fibroblasts proliferation were studied. Results on fibroblasts’ adhesion were considered as an index of matrix efficacy to favor wound healing in proper times. Furthermore, the ability of CS/HA matrices to prevent bacterial adhesion and biofilm formation was evaluated by using *Staphylococcus epidermidis* as the model microorganism. 

## 2. Results and Discussion

This work comes under the scope of the scientific researches striving to better understand the role of biopolymers in controlling tissue regeneration processes and healing of chronic wounds. In this context, chitosan and hyaluronic acid were the object of the investigation in this study due to their interesting biological properties. Specifically, these two biopolymers were blended at different weight ratios to obtain CS/HA 2D-matrices (films), at increasing HA content (Table 1), potentially applicable as wound dressings.

The obtained films were characterized in terms of physico-chemical properties and biological performance. In particular, besides studying the ability of CS/HA films to favor in vitro human fibroblasts’ adhesion and proliferation, bacterial fouling on the films was also investigated given the common infectious complications associated with chronic wounds.

### 2.1. Physico-Chemical Characterization of CS/HA 2D-Matrices

**Figure 1:**
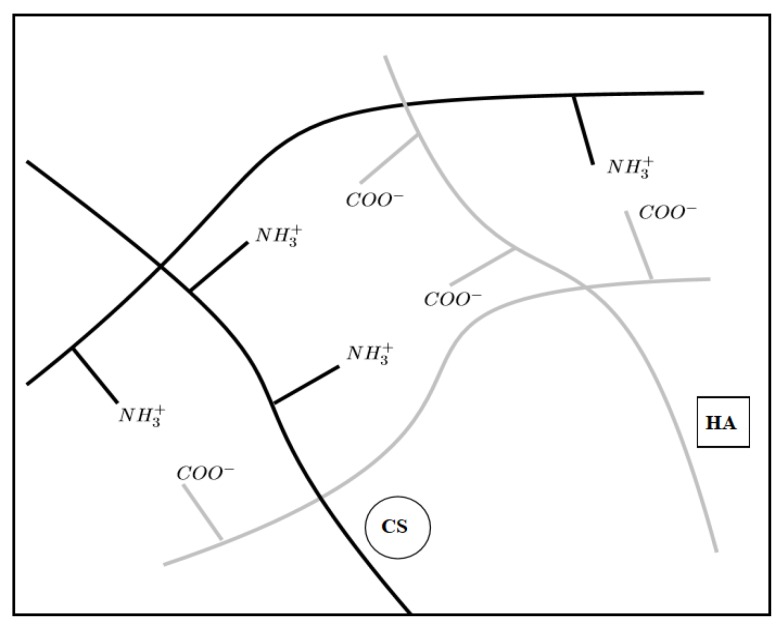
Scheme of electrostatic interactions between CS amino groups and HA carboxyl groups.

CS and HA can interact by establishing electrostatic interactions between CS amino groups and HA carboxyl groups (ionotropic gelation) as schemed in Figure 1.

**Figure 2:**
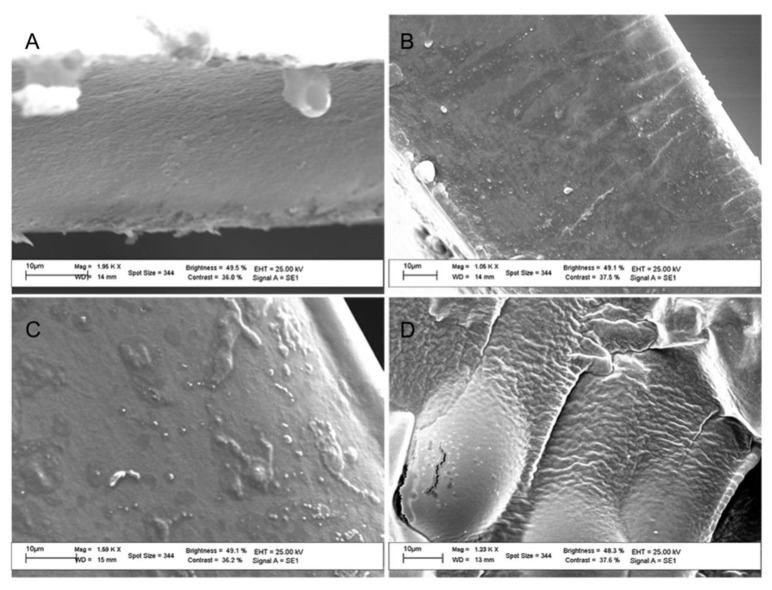
SEM images of CS (**A**), CS/HA5 (**B**), CS/HA25(**C**) and CS/HA35 (**D**) films.

The compatibility of the blends was evaluated by SEM observations of the morphology of fractured CS/HA films. The cross-sectional SEM images of selected samples are displayed in Figure 2. Pristine CS film showed a homogeneous structure that was partially modified by HA incorporation. Indeed, in the blended membranes, a granular substance was observed already at 5% HA content (Figure 2B). Such granular substance formed clusters of significant size at higher HA content (Figure 2C), suggesting a partial incompatibility of the two biopolymers. A peculiar structure was instead showed by the CS/HA35 sample, where a high roughness was observed. The presence of aggregates in CS/HA blends was also reported by Xu et al. who studied by atomic force microscopy (AFM) the morphology of blends with HA weight ratios of 0.1%, 0.25% and 0.5%. The authors considered such aggregates, whose size enlarged with the increase in HA amount, as the polyelectrolytic complex of CS and HA [30].

**Figure 3:**
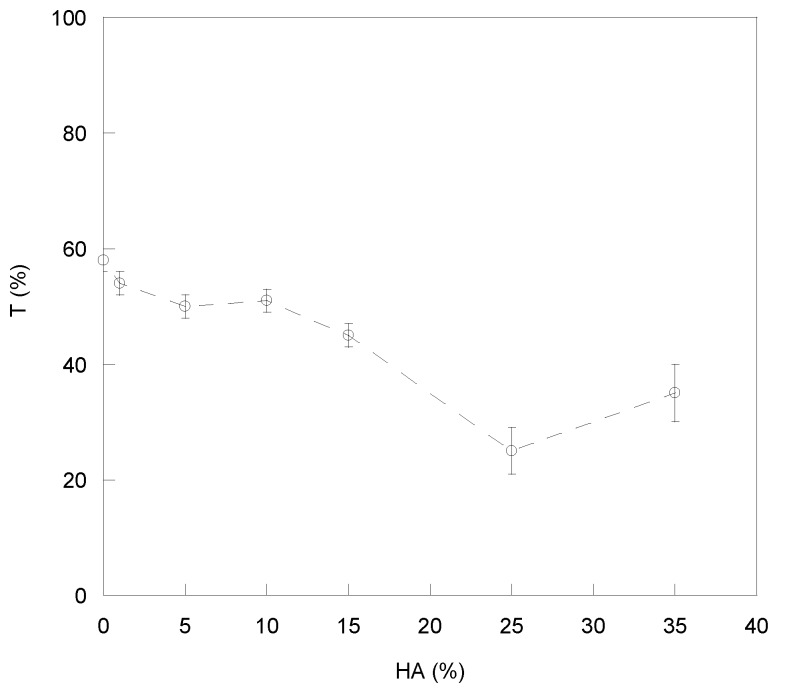
Optical transmittance (T%) of prepared films versus the HA amount.

The presence of aggregates in the blends affected transparency of films as shown in Figure 3, where transmittance (T) was reported as a function of HA percentage. 

Indeed, all of the CS/HA matrices showed a T% value lower than pure CS. However, the reduction in T% was significant only for HA contents higher than 10%, suggesting a good compatibility between CS and HA up to 10% HA. Such CS-HA compatibility was compromised at higher HA contents in agreement with SEM observations.

**Figure 4:**
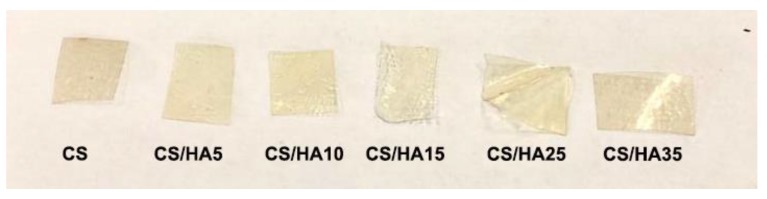
Photos of CS and CS/HA 2D-matrices.

Notwithstanding that, all of the matrices maintained a transparency sufficiently good to permit, if used as wound dressings, the visual control of the wound healing progress without dressing removal (Figure 4).

**Figure 5:**
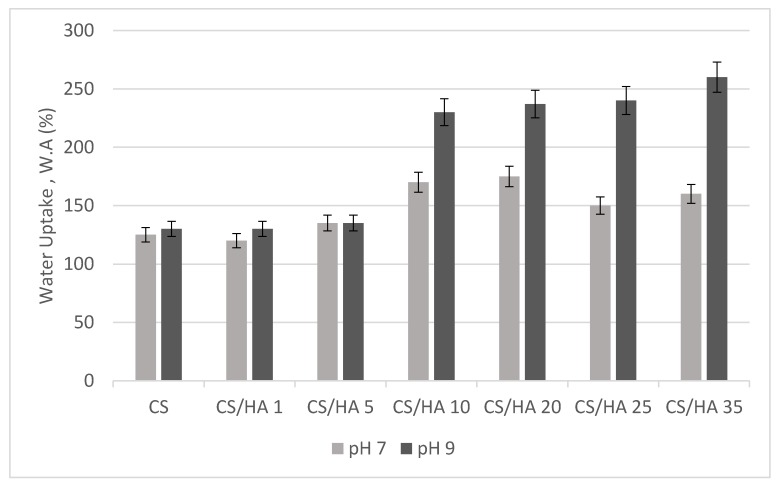
Equilibrium water uptake of CS and CS/HA matrices in PBS (pH 7) and Tris (pH 9) buffer.

An important requirement for wound dressings is the ability to absorb wound exudates ensuring both a good structural integrity and gas permeability. To this aim, film water uptake (WA), soluble fraction (SF) and water vapor transmission rate (WVTR) were investigated. In Figure 5, the equilibrium water uptake values for CS/HA matrices in PBS buffer (pH 7) and Tris buffer (pH 9) were reported. The study at alkaline pH was performed on the basis of the knowledge that chronic wounds, like venous leg ulcers and pressure sores, have alkaline pH for most of the time, except for the re-epithelization phase [36].

As it can be seen in Figure 5, at both pH values, CS/HA matrices, except from CS/HA1 and CS/HA5, showed a degree of water uptake higher than pristine CS and this property grew with HA increasing in the matrix. Reasonably this trend was related to the well-known HA hydrophilicity and water retention capacity. In addition, a possible partial deprotonation of HA carboxylic groups at basic pH resulted in matrix equilibrium WA values higher than at neutral pH, suggesting the possibility of using these dressings for chronic wounds where an alkaline pH has been detected for most of the time [36].

Interestingly, after swelling, all of the matrices kept a good structural integrity, as demonstrated by the low soluble fraction of the matrices, in all cases less than 2%, presumably related to CS–HA electrostatic interactions.

In agreement with these findings, also film wettability, evaluated by contact angle (CA) analysis, improved with HA, as shown in Table 1. Particularly, the films with HA contents higher than 10% showed a moderate hydrophilicity with contact angles ranging from 84° of CS/HA15 to 72° of CS/HA35. Such moderate wettability could be advantageous for material biocompatibility since a suitable surface hydrophilic/hydrophobic balance is needed to have a proper cell–material interaction [37].

The rate of water vapor transmission (WVTR) is also an important dressing feature representing its ability to retain moisture. As it can be observed in Table 1, at low HA contents (1%, 5% and 10%) matrix WVTR was higher (595, 672 and 624 g/day·m^2^) than pristine CS (576 g/day·m^2^). In contrast, WVTR decreased for HA contents higher than 10%, reaching a 430 g/daym^2^ value in the CS/HA35 sample. These findings are presumably related to the ability of HA to retain water molecules. A decrease in WVTR with HA increasing was also reported by Xu et al. [30]. In general, dressings should guarantee a good transpiration to maintain an optimal moisture level in the wound. A dressing with an extremely high WVTR may lead to wound dehydration, while a dressing with a too low WVTR may cause the accumulation of wound exudates. There is not a unique WVTR value required for a dressing since this parameter strongly depends on the type of wound as well as on the wound healing stage [38]. Notwithstanding that, several studies [39,40] report a WVTR value of 2000–2500 g/m^2^·day as optimal to maintain good moisture in the wound bed. On this basis, our matrices could be used essentially for low or moderately exuding wounds, where a high WVTR is inappropriate because causing dressing adhesion to the wound bed with consequent pain and trauma on the removal phase [41].

**Figure 6:**
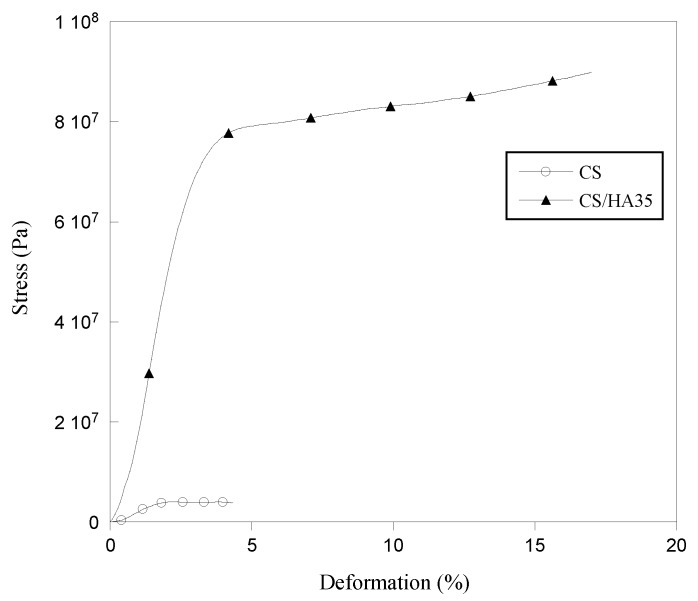
Stress–strain curves for CS and CS/HA35 matrices.

The mechanical behavior of CS and CS/HA matrices was also investigated by performing tensile tests. Generally, soft and flexible dressings are recommended because they can provide easy application and removal. In Figure 6, the stress–strain curves of pure CS and CS/HA matrix at higher HA content (35%) are reported while in Table 1 the values of Young Modulus (E), tensile strength at break (TS) and elongation at break (EB) are reported for all of the samples.

Interestingly, the addition of HA in CS films increased both the tensile strength and elongation at break giving matrices more elastic and in the same time more resistant than pure chitosan. Specifically, elongation at break of CS/HA matrices increased by ca 40% compared to CS when HA was in the 5–15% range, while tripled for HA ranging from 25% to 35%. Probably, the increase in TS, already significant at low HA concentration (5%), was related to the formation of polyelectrolytic complexes between CS and HA, which created a reinforcing ionic network. The elongation at break was found to be high at 25% and 35% HA contents, presumably because of the presence of HA/CS clusters immersed in a CS matrix, which provided elasticity.

### 2.2. Biological Characterization of CS/HA 2D-Matrices

**Figure 7:**
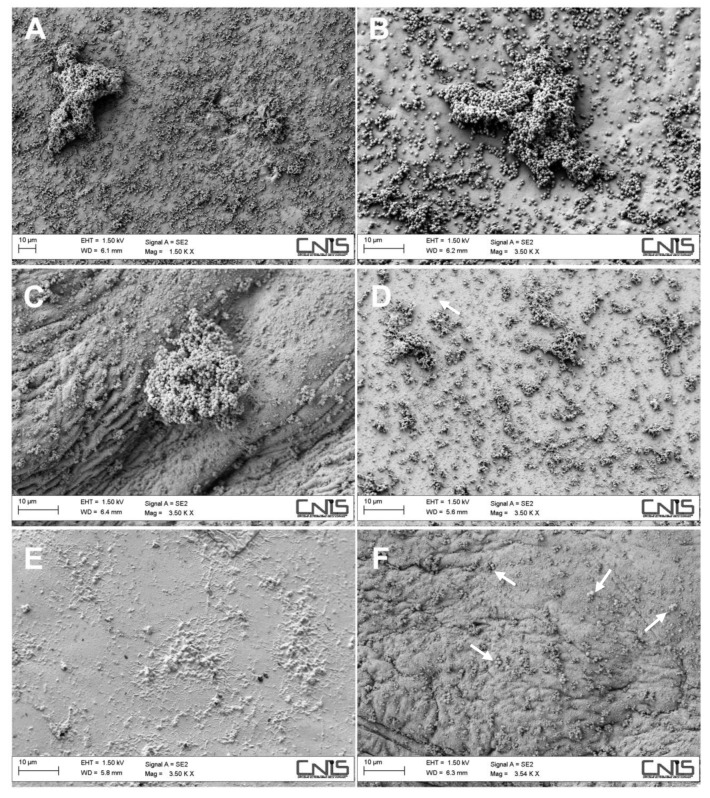
SEM micrographs of bacterial cells adhered onto CS at two magnifications (**A**,**B**), CS/HA1 (**C**), CS/HA5 (**D**), CS/HA10 (**E**) and CS/HA15 (**F**) matrices. White arrows indicate bacterial cells.

To evaluate the possible activity of CS/HA matrices in interfering with microbial adhesion and biofilm formation, the matrices were challenged with *S. epidermidis*, an opportunistic bacterial species often involved in wound infections. After incubation with bacterial suspension for 24 h, bacteria adhered to the dressing surface were fixed for SEM observations. Experiments were not performed on the CS/HA25 and CS/HA35 samples because they dissolved under the adopted experimental conditions. In Figure 7, SEM micrographs showing the bacterial adhesion onto the different surfaces are reported.

As it can be observed, a heavy bacterial colonization on pure CS film was observed with the presence of both single colonies and large biofilm structures (Figure 7A,B). The CS/HA1 matrix still showed bacterial aggregates of significant size (Figure 7C). In contrast, CS/HA matrices with HA contents 5%, 10% and 15% were essentially free from *S. epidermidis* colonization, with just the presence of few sporadic adhering cells and lots of residues of damaged bacterial cells. Possibly, an influence of surface wettability (contact angle) onto bacterial adhesion can be hypothesized. Indeed, it is generally accepted that hydrophilic surfaces are less prone to bacterial adhesion due to hydration effects [42,43,44]. Bacteria cell count reported in Table 1 confirmed SEM observations.

**Figure 8:**
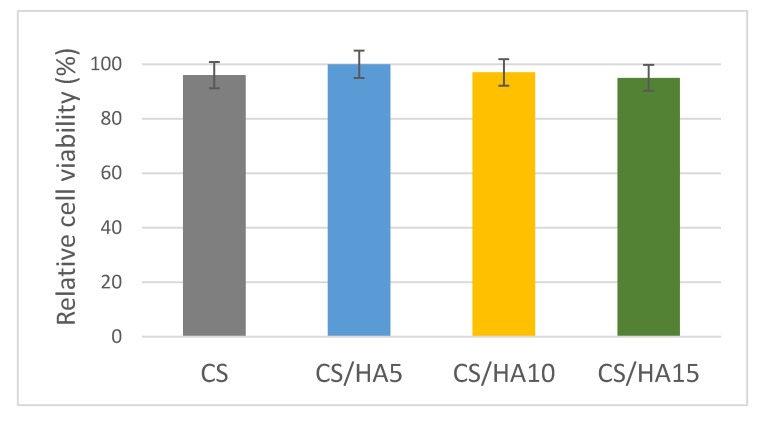
Fibroblasts viability on pure CS and CS/HA matrices over a 7-day period.

Finally, the adhesion of fibroblasts onto pure CS and CS/HA matrices was investigated as an index for potential use of CS/HA matrices in favoring wound healing. The CS/HA1 matrix was not tested because of the negative results obtained in the bacterial adhesion experiments. Fibroblasts viability onto CS/HA matrices was assessed by MTS-based colorimetric assay. In Figure 8, the fibroblasts viability on pure CS and CS/HA matrices over a 7-day period is reported. As it can be observed, good cell viability was found for all of the tested matrices.

**Figure 9:**
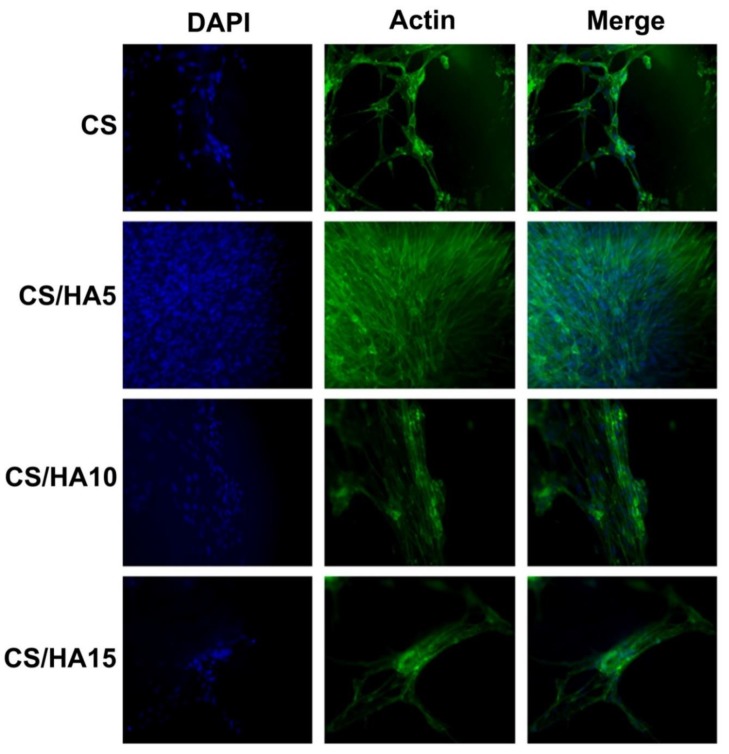
Immunofluorescence detection of fibroblasts grown onto CS, CS/HA5, CS/HA10 and CS/HA15 films. Bar = 100 µm.

Fibroblasts’ adhesion and proliferation was evaluated with immunofluorescence tests and microscopic observations (Figure 9). 

As it can be observed, fibroblasts maintained their morphology on all of the matrices, confirming a good biocompatibility and non-toxicity for all prepared samples. The evaluation also suggested that the adhesion property was related to the amount of HA present in the samples. In particular, the best results were obtained for the matrix having a 5% HA content, which retained the higher number of cells. The increase in HA concentration slightly reduced cell retention. Probably, the greater matrix hydrophilicity (see contact angle, Table 1) influenced the fibroblasts adhesion process. This evidence was already reported in the literature. Indeed, Deng et al. [45] studied the post-operative cell adhesion onto CS/HA injectable gels suggesting that an excessive hydrophilicity could compromise the cell adhesion process.

Furthermore, despite pure CS and CS/HA5 showed similar wettability (Table 1), fibroblasts adhesion was greater onto the CS/HA5 matrix than pure CS. Presumably, the introduction of HA caused a decrease in polymer charge density, with benefits for cell adhesion [46]. Overall, obtained findings indicate how HA is able to improve the adhesion of fibroblasts even if this property seems to be concentration-dependent. In fact, on one hand an enhanced wettability (low CA) improves fibroblasts cell adhesion while on the other hand it may compromise cell–cell interactions and cell retention [47,48].

## 3. Materials and Methods

### 3.1. Preparation of Chitosan–Hyaluronic Acid 2D-Matrices

Chitosan (medium molecular weight, 200–800 cP at 1 wt % in 1% acetic acid at 25 °C, 75%–85% deacetylated, Sigma Aldrich, Darmstadt, Germany) was dissolved in 1% acetic acid at a 2% wt/V concentration and dialyzed (membrane cutoff 3.5 kDa) for 24 h before use. Then, CS solution was mixed with hyaluronic acid (M_w_ 10.000–30.000, Sigma Aldrich) solution (0.5 wt%/V) in volumes such to obtain the following HA concentration: 1%, 5%, 10%, 15%, 25% and 35%. Continuous stirring was performed up to obtain viscous and homogeneous mixtures. After mixing, HA/CS solutions were casted onto Petri dishes (diameter 8 cm) and the solvent was evaporated for 24 hr at room temperature. Two dimensional matrices with a 100 µm average thickness were obtained. According to the HA content, samples were named CS/HAx where x is the HA percentage in the polymer matrix.

### 3.2. SEM Observations

To have information on the compatibility of the two blends, the bulk of the films was observed by field emission scanning electron microscopy (FESEM, AURIGA Carl Zeiss AG, Oberkochen, Germany). For the analysis, films were fractured by immersion in liquid nitrogen, and the fractured surface was gold sputtered and observed.

### 3.3. Film Transparency 

The optical transparency of films was studied by UV-vis spectroscopy according to the ASTM D1746-09 recommendations. Specifically, film samples were cut into rectangles and placed in a spectrophotometer cell. The absorbance spectrum (420–640 nm) was recorded for each sample using a diode array spectrophotometer (HP8452A, Hewlett Packard, Palo Alto, CA, USA). Absorbance value at 550 nm wavelength was used to calculate the transmittance percentage (T%), the index of film transparency, according to the following equation:

(1) $$A=\log\frac{100}{T\%}=2-\log T\%$$

For each sample, the analysis was repeated five times.

### 3.4. Water-Uptake Capacity and Soluble Fraction

Water uptake (WA) of films was determined by immersing weighted films into phosphate (PBS) buffer (pH 7.4) or TRIS buffer (pH 8) at room temperature for 24 hr where a WA plateau was reached. Following immersion, films were removed from water and weighed (W_f_), after removal of the excess of solvent using filter paper. Water uptake was defined as follows:

(2) $$WA\left(\%\right)=\frac{W_{f}-W_{o}}{W_{0}}\times 100$$

where W_f_ is the weight of the sample at 24 hr swelling and W_0_ is the initial weight of the film. Three parallel swelling experiments were performed for each sample and data were reported as average value ± standard deviation.

For soluble fraction (SF) determination, 24 hr-swollen films were dried in an oven until constant weight. SF was measured by the differences between the initial weight (W_0_) and the weight of dried W_e_ films as reported in the following equation:

(3) $$SF=\frac{W_{o}-W_{e}}{W_{0}}\times 100$$

Each reported value was the mean of 3 measurements.

### 3.5. Water Vapor Transmission Rate Test 

The water vapor permeability test of films was carried out using the ASTM E96 procedure (Standard Test Methods for Water Vapor Transmission of Materials). Briefly, films were sealed as a patch onto a glass container (diameter 5.5 cm and height 3.5 cm) filled with 10 mL of water. The assembled system was weighted at different times and the water vapor transmission rate (WVTR), expressed as (g/day·m^2^), was calculated from the slope of the straight line obtained by plotting the following equation:

(4) $$WVTR=\frac{W}{t\times S\;}$$

where W is the weight, expressed in grams, collected at each time (t), expressed in days and S is the exposure surface area (m^2^).

### 3.6. Static Contact Angle Measurement

To examine the film wettability, the static water contact angle was measured at room temperature by the drop method. Specifically, a water droplet (Milli-Q water) was deposited on the film surface and a picture was taken. The obtained image was then elaborated with software SigmaPlot (Systat Software Inc., San Jose, CA, USA) to define the drop base length (D) and the drop height (h). The contact angle was determined as follows:

(5) $$\vartheta {}^{\circ}=2\;\tan^{-1}\frac{2\;h}{D}$$

Each reported contact angle was the mean value of 3 measurements.

### 3.7. Mechanical Characterization

Mechanical properties of the CS/HA matrices at different HA content were studied by tensile tests using an ISTRON 4502 instrument (Instron Inc., Norwood, MA, USA). For the analysis, films were cut into rectangular specimens (7 cm × 1 cm × 0.1 mm) and fixed between the two Instron flat plates. A constant deformation rate of 1 mm/min was set while a 10 N load cell was used. 

### 3.8. Evaluation of Staphylococcus epidermidis Adhesion onto CS/HA Matrices

Bacterial adhesion onto CS/HA matrices was evaluated by FESEM observations and bacterial cell count. *S. epidermidis* (ATCC 35948) was used as model microorganism due to its common involvement in wound infections. Squared specimens (1 cm × 1 cm) of pristine CS matrix and CS/HA matrices were immersed in a Tryptic Soy Broth (TSB) bacterial suspension (1.5 mL) at a 10^8^ CFU/mL concentration (optical density = 0.1 at 625 nm). After incubation for 18 h at 37 °C, polymer specimens were collected, washed three times with PBS buffer and submitted either to a cell count or to fixation for SEM observation.

As far as a bacterial cell count is concerned, after incubation polymer specimens were put into test tubes with 10 mL of phosphate buffer, and sonicated for 5 min to remove the adherent cells. Six 10-fold dilutions were prepared, and three 10 μL aliquots of each dilution were plated onto TSA plates. Plates were then incubated at 37 °C for 18 h, and colony forming units (CFUs) were counted and referred to the matrix surface unit (CFUs/cm^2^).

As far as SEM observation is concerned, specimens were treated with 2.5% glutaraldehyde in 0.1 M cacodylate buffer (pH 7.4) at room temperature for 30 min, dehydrated through graded ethanol, treated with hexamethyldisilazane for 20 min and gold-sputtered for FESEM observation. 

### 3.9. Cell Culture 

Human dermal primary fibroblasts obtained from adult male patients, isolated as previously described [49], were used. Cells were cultured at 37 °C and 5% CO_2_ in Dulbecco’s modified Eagle’s medium (DMEM) supplemented with 10% fetal bovine serum (FBS), 1% penicillin/streptomycin, 1% L-glutamine, 1% Na-pyruvate and 1% non-essential amino acids (Sigma-Aldrich, Co. Saint Louis, MO, USA).

#### 3.9.1. Sample Preparation

Before plating, each polymer matrix was conditioned in culture medium for 2 hours to avoid variation potentially toxic for cells. After conditioning, cells were plated at a density of 8 × 10^3^ cells/film and cultured for either 7 days or 15 days.

#### 3.9.2. Cell Viability

Cellular viability was quantified after 7 days of incubation by measuring the mitochondrial dehydrogenase activity using the dye 3-(4,5-dimethylthiazol-2-yl)-5-(3-carboxymethoxyphenyl)-2-(4-sulfophenyl)-2H-tetrazolium (MTS); Promega Corporation, Madison, WI, USA) based colorimetric assay. Briefly, after 7 days, 20% (*v/v*) of the MTS dye was added in the culture media and cells were cultured for 4 h to allow the formation of soluble formazan crystals by viable cells. Spectrophotometric absorbance was measured at 490 nm using a multi-plate reader Appliskan (Thermo Fisher, Waltham, MA, USA). Cells cultured in the absence of the CS/HA matrices were taken as a control.

#### 3.9.3. Immunofluorescence

To visualize actin filaments an immunofluorescence analysis was performed. Cells were cultivated 15 days onto 2D-matrices after, then they were washed in PBS, fixed in 4% paraformaldehyde in PBS for 15 min at 4 °C and permeabilized with 0,5% Triton-X100 in PBS for 10 min at room temperature. After blocking with 3% bovine serum albumin (BSA) in PBS for 30 min at room temperature cells were incubated with Phalloidin Alexa Fluor 488 (Immunological Sciences, Rome, Italy) 1:40 for 20 min at room temperature. Cells were ultimately washed in PBS and incubated with 4′,6-diamidino-2-phenylindole (DAPI, Invitrogen, Thermo Fisher Scientific, Massachusset, USA) to visualize the nuclei and analyzed with Leica DM microscope (Leica Microsystem, Milan, Italy). 

### 3.10. Statistics

Analysis of variance comparisons were performed using Mini-Table Differences, which were considered significant for *p* < 0.05. Data are reported as means ± SD.

## 4. Conclusions

Chitosan–hyaluronic acid bioactive matrices for wound dressings were fabricated. The incorporation of HA in CS matrix affected matrix physical properties in a concentration-dependent manner. Specifically, at all of the tested HA concentrations (1–35%) a decrease in film transparency was observed, possibly related to the formation of CS/HA polyelectrolytic aggregates. In contrast, an increase in water uptake and surface wettability (CA) was observed only for a HA content greater than 10%. HA increased matrix WVTR at low contents (1% and 5%), possibly because it interfered with CS structure, but hampered water vapor permeability at higher concentrations due to its water retention capability. Interestingly, the addition of HA in CS films, already at low concentration, affected also mechanical properties providing matrices more resistant and flexible than pure CS with benefits in terms of wound care.

The biological characterization of the matrices gave encouraging results in the perspective of using them for wound management. Indeed, *S. epidermidis* adhesion onto the CS/HA matrices was significantly reduced compared to pure CS, expect from CS/HA1. That behavior confirms the initial hypothesis that HA at significant concentrations (equal to or greater than 5%) could provide the dressing with intrinsic antibacterial fouling properties. Therefore, CS/HA dressings can avoid the use of antibacterial agents for preventive purposes. Fibroblasts adhesion also benefited from HA presence in the film, especially at 5% content, where a good compromise of surface charge and hydrophilic/hydrophobic balance was presumably achieved. Therefore, the CS/HA5 matrix seems to combine, better than the others, good physical performance (the greatest WVTR, good water uptake and suitable transparency) with enhanced biological properties.

## Figures and Tables

**Table 1:** Acronyms and properties of CS/HA matrices. E = Young modulus; TS = Tensile strength; EB = Elongation at break. WVTR = water vapor transmission rate. ND = Not Detectable (<5 × 10^3^ CFU/cm^2^); NP = Not Performed (because of film dissolution in the adopted experimental conditions).

Sample	HA Content (%)	Contact Angle (ϑ°)	Mechanical Properties			WVTR (g/day m^2^)	*Staph. ep.* CFUs/cm^2^
			E (GPa)	TS (MPa)	EB (%)		
**CS**	0	98 ± 3	0.2 ± 0.04	5.0 ± 1	5 ± 1	576	9 × 10^7^
**CS/HA1**	1	96 ± 2	0.2 ± 0.03	4.3 ± 0.5	6 ± 1	595	4 × 10^7^
**CS/HA5**	5	93 ± 1	0.5 ± 0.1	40 ± 8	7 ± 1	672	ND
**CS/HA10**	10	89 ± 2	2.0 ± 0.4	60 ± 12	7 ± 1	624	4.8 × 10^4^
**CS/HA15**	15	84 ± 1	1.5 ± 0.3	40 ± 7	8 ± 2	500	2.5 × 10^4^
**CS/HA25**	25	82 ± 2	2.0 ± 0.2	60 ± 10	13 ± 2	456	NP
**CS/HA35**	35	72 ± 5	4.0 ± 0.5	100 ± 15	16 ± 3	432	NP
